# Protocol for Rescuing Young Cassava Embryos

**DOI:** 10.3389/fpls.2020.00522

**Published:** 2020-05-08

**Authors:** Zaida Lentini, Geraldine Restrepo, María E. Buitrago, Eddie Tabares

**Affiliations:** Center of Specialized Natural and Biotechnological Ingredients (CINEB), School of Natural Sciences, Universidad Icesi, Cali, Colombia

**Keywords:** cassava, embryo rescue, immature zygotic embryos, inter-specific crosses, wide crosses

## Abstract

Embryo rescue (ER) in cassava breeding has several relevant applications, from the recovery of broad crosses to the recovery of seeds from the standard pollination program. Cassava fruit setting may drop from 100%, during the 1st week after pollination, to less than 40% during the 2nd week after pollination due to the abscission of fruits depending on genotypes. Therefore, the availability of an ER protocol for early stages of embryo development, in particular during the first 2 weeks after pollination (prior the cotyledonary stage), could have practical implications for cassava breeding. Until now, attempts to recover cassava immature embryos at stages of development earlier than the cotyledonary stage failed. The earliest successful rescue reported in cassava is from embryos excised 32–36 days after anthesis (DAA). However, limited information was available regarding embryo development in cassava. This work studied and documented the stage of embryo development in histological sections of hand-pollinated ovules fixed from 1 to 30 days after anthesis (DAA). At 7 DAA, zygotes were just at the first stages of cell division (pro- embryo stage). At 14 DAA, embryos were at the pre-globular stage. Embryos at the early globular stage were observed in sections fixed at 21 DAA, and at the proper globular stage at 24 DAA. Samples at 30 DAA contained cotyledonary embryos that easily developed after ovule culture into viable plants using existing protocols. A second contribution of this work is the development of a protocol for the recovery of fully developed plants from immature embryos rescued and cultured *in vitro* as early as 7–14 DAA. Since embryos collected at this age are at the pro-embryo to pre-globular stage, ovary/ovule culture was necessary. A method is described whereby ovules were cultured to allow the development of pro-embryos and pre-globular stage embryos into the cotyledonary stage. Subsequently, these mature embryos were excised from the ovules to induce germination and the recovery of fully developed plants.

## Introduction

Cassava (*Manihot esculenta* Crantz), a perennial shrub, is one of the most important calorie-carbohydrate sources in the tropics, adapted to a broad range of environments due to its tolerance to drought and acid soils (Kawano, 2003; Ceballos et al., 2004, 2012). This main staple food for subsistence farming has become an important raw source for industrial applications as well. Cassava is the second most important source of starch worldwide (Karlstrom et al., 2016). Cassava breeding is difficult compared to other crops, mainly due to the highly heterozygous nature of progenitors (Ceballos et al., 2015) and its inbreeding depression (Rojas et al., 2009; Wang et al., 2011). Cassava breeding is also slow because of the low multiplication rate. There is a need to increase genetic gains and to widen the genetic base in cassava (Ceballos et al., 2012, 2015, 2016).

Embryo rescue (ER) is one of the earliest and successful forms of *in vitro* culture techniques used to assist the development of plant embryos that might not survive to become viable plants. This technique nurtures the immature or weak embryo, thus allowing it to survive. ER techniques have many significant applications in plant breeding, as well as basic studies in physiology and biochemistry (Collins and Grosser, 1984; Ramming, 1990; Bridgen, 1994; Sharma et al., 1996; Haslam and Yeung, 2011). Zygotic embryos have been successfully rescued, at younger (promebryos) or older (mature embryos) stages of development. The technology has been used in about 100 different species from both temperate and tropical climates, comprising crops, fruit, and forest trees as well as wild species (Haslam and Yeung, 2011).

Embryo rescue is particularly attractive for recovering plants from sexual crosses where the majority of embryos cannot survive *in vivo* or become dormant for long periods. The most widely used ER has been for obtaining plants from hybridizations in which the endosperm does not develop appropriately causing embryo abortion (Reed, 2005). The success of the ER relies on providing a proper substitute for the endosperm through the artificial nutrient medium, hence allowing the embryo to continue its development. ER procedures have been very successful in overcoming barriers to wide hybridization in a broad range of plant species (Collins and Grosser, 1984). ER plays an important role in modern plant breeding, allowing the development of interspecific and intergeneric crop hybrids. These types of crosses would normally produce seeds that eventually abort. Wide crosses can result in small shrunken seeds, which indicate that fertilization has occurred, however, the seed fails to develop. Many times, distant hybridizations fail to undergo normal sexual reproduction, thus ER can also assist in circumventing this problem.

In the case of cassava, ER may be useful for the recovery of seeds from hand-made pollination, which could be low depending on genotypes (Yan et al., 2014). ER may also play an important role in modern cassava breeding, allowing the development of hybrids from broad crosses. ER was first reported in cassava for *in vitro* germination of mature embryos 60 days after open-pollination (DAP) (Biggs et al., 1986; Fregene et al., 1999). A similar protocol was used to develop and multiply a backcross population of cassava from hybridization with *Manihot esculenta* ssp. *flabellifolia* (Akinbo et al., 2010). In this case, the embryos were advanced in their development (excised 40 DAP), which was supported by the presence of normal endosperm (Akinbo et al., 2010). Embryo rescue was used in interspecific pollination with castor bean (*Ricinus communis*) in early attempts to produce doubled haploids (Ramos-Abril, 2018; Baguma et al., 2019). Yan et al. (2014) suggested to rescue embryos at earlier stages, from 32 to 36 DAP, when most embryos are at cotyledonary stage, the endosperm is already formed, and the embryos are visible and easier to excise from the pollinated ovules without injury. At 38 DAP or beyond, seeds are too hard and embryo may be injured during the excision process.

In cassava, the availability of an ER protocol for early stages of embryo development, in particular during the first 2 weeks after pollination (prior the cotyledonary stage), could not only have an important application for broad crosses, but also for the standard pollination program. One of the mayor bottlenecks that cassava breeding faces is that fruit setting may drop from 100%, during the 1st week after pollination, to less than 40% during the 2nd week after pollination due to the abscission of fruits (Yan et al., 2014). Apparently, the embryo and endosperm development in cassava is remarkably slow till the 4th week after pollination (Yan et al., 2014). Various other factors may also explain the abscission of fruits after pollination including genotype differences, biotic and abiotic stresses, and ploidy conditions. Similar trends were reported for crosses with *Manihot esculenta* ssp. *flabellifolia* (Akinbo et al., 2010) or for wide crosses with *R. communis* (Ramos-Abril, 2018; Baguma et al., 2019). Therefore, the availability of an embryo rescue protocol in cassava, prior to the 2nd week after pollination, is highly relevant. Until now, attempts to recover cassava immature embryos at stages of development earlier than the cotyledonary stage failed. Yan et al. (2014) reported that the use of GA_3_ in the M6 culture medium could probably promote the development of immature embryos. They also found that immature embryos without visible cotyledons were too small to germinate. Younger embryos may require the inclusion of other tissue such as embryo sac and even part of the ovary in order to sustain full development (Yan et al., 2014).

The two most important aspects of zygotic embryo culture are: (1) the composition of the culture medium, and (2) the excision of the embryo to be cultured (Haslam and Yeung, 2011). However, prior to culturing, it is important to define the target developmental stage of the embryos, which depends on the aim of the study. With embryo rescue, for example, it is important to know when they begin to abort so that dissections are carried out prior to abortion. If necessary, histological sections can be used to determine the stages of development of embryos, which also will determine the composition of medium to be used (Haslam and Yeung, 2011). Establishing a developmental timetable is thus extremely useful. Although creating the timetable is time consuming, it greatly facilitates subsequent embryo collection at the desired stage of development. Taking into account the high rate of abortion in cassava during the first 2 weeks after pollination (Yan et al., 2014), a protocol for the rescue of immature zygotic embryos during that period is desirable. It is worth emphasizing the limited information available in the literature regarding the development of embryos in cassava after pollination, particularly from zygote formation through the globular and early cotyledonary stages of development.

The composition of medium used to sustain embryos is key for a successful culture. It is well known that the optimal composition of the medium changes during embryonic development. Generally, the younger the embryo, the more complex its nutritional requirements are. As they mature, embryos can be grown in a simpler inorganic salt media (Yeung et al., 2001). Thus, the most important aspect of embryo culture work is the selection of medium that meet the constantly evolving needs of isolated, growing embryos. Although there are a number of medium formulas in use, many have not been systematically tested.

The research reported here was aimed at developing a protocol for recovering cassava plants from embryos cultured as early as the first two weeks after anthesis. The specific objectives of the work were; (1) To assess embryo development in cassava from 1 to 30 days after anthesis (DAA); and (2) To develop a protocol for the recovery of fully developed plants from ovules cultured within the first 2 weeks after pollination. In this work, we present an alternative approach for embryo rescue in cassava that takes into account the different requirements that young embryos have and the complexity for simulating a proper environment for their development.

## Materials and Methods

### Plant Material and Growth Conditions

The cassava elite line SM1219-9, used by the cassava-breeding program at the International Center of Tropical Agriculture (CIAT) was selected because of its profuse flower production. This genotype starts flowering 5–6 months after planting and embryo development in the three loculi of the same fruit is relatively uniform compared with other genotypes (Yan et al., 2014). Field nurseries were grown at CIAT Experimental Station in Palmira (Colombia). The soil is a clay loam, pH 7.2, and contains adequate levels of macro- and micro-elements for cassava growth and development. Phytosanitary conditions at the station are optimal, requiring minimum or no chemical applications for insects and disease control. Herbicides were applied before planting and for a short time thereafter. Mean daily temperature was 27°C during the day and 18°C at night. Plants were rain-fed and irrigated when required. Cassava inflorescences are cyathia (singular cyathium), one of the specialized pseudanthia (“false flowers”) forming the inflorescence of plants in the genus *Euphorbia* (*Euphorbiaceae*). In cassava, cyathia structures are reduced to a single style wrapped in 5 petal-like bracts. The female cyathium has a trilocular ovary (three carpels), and each loculus contains one ovule. Female cyathia from the third or fourth flowering events of healthy-looking and vigorous plants, with profuse cyathia formation of similar morphology and developmental stage, were used for the experiments.

### Determination of Embryo Developmental Stages

Cassava had not been the subject for histological studies on embryo development until now. Because of the limited information available, it was necessary to carry out a preliminary study that would allow predicting the stage of development of embryos when carpel/ovule culture was initiated. In this work, day after anthesis (DAA), rather than day after pollination (DAP) was used as reference for the establishment of the cassava zygotic embryo developmental timetable. Ramos-Abril et al. (2019) reported that about 74% of the seeds produced by SM1219-9, the genotype used in this work, from open pollinations took place at anthesis day, 24% at 1 DAA and only 2% at 2–3 DAA. In other words, in this genotype, 98% of the seeds produced from open pollinations is from the anthesis day or from 1 day later. Moreover, stigmas dehisce from pistils typically 4 DAA, thus pollinations cannot occur later than 3 DAA. In the worst-case scenario, therefore, 100% pollination in open-pollinated SM1219-9 occurs within 4 DAA. Therefore, it was assumed that day of pollination and anthesis day were the same or very similar. This assumption was taken into account for the experimental design of this work, the interpretation of the results and their corresponding discussion, and hence, this better-controlled condition was feasible.

Hand-pollinated cyathia were used to assess the stage of development of embryos at different times after anthesis. Anthesis of female cyathia occurs early in the afternoon, and it is easy to predict those cyathia that will open within a day. Cyathia were selected, marked and covered with mash bags the day before anthesis or early on the anthesis day (7:00–9:00 am at the latest), and before bracts opened to avoid accidental pollination. The isolating bags remained on the cyathia after hand-pollinations had been made and until sample collection.

The cyathia were collected 1, 2, 3, 7, 14, 21, 24, or 30 DAA. Fifteen cyathia per date (45 ovules each) were analyzed. Cyathia were collected always at 1:00–2:00 pm, placed in zip-plug bags within a styrofoam cooler with refrigerant gel, and transported to the laboratory within the next hour after collection. Samples were kept cool for 1–2 h, until fixed in FAA [38% formaldehyde (Sigma-Aldrich F8775): glacial acetic acid: (Sigma-Aldrich 537020): 75% ethanol; 7:5:88, v/v)] at least for 7 days. Then, samples were washed for a minimum of 24 h in 70% ethanol to eliminate most of formaldehyde and acetic acid, and stored in 70% ethanol at 4°C until further use. Samples were then either sectioned with a microtome or a vibratome.

Samples sectioned with a microtome were dehydrated in a gradient of absolute ethanol: tertiary butanol (Sigma-Aldrich 471712) in 7 steps of 1,5 h each, ending with pure tertiary butanol. Then, sections were embedded in paraffin blocks according to Ruzin (1999). Longitudinal, 4 μm or 10 μm thick, sections (depending on ovaries age) were made with an American Optics Spencer 820 Rotary Microtome. Sections were placed on microscope slides, dried at 60°C for at least 1 h, and then stained with Safranin O - Fast Green (Fernandez-Luqueno et al., 2008). Samples were analyzed under light and dark field illumination with a Nikon Eclipse 55i microscope. Photographs were taken with a high-resolution Nikon DS-Fi1 5-megapixel cooled color digital microscope camera, coupled with a NIS-Elements imaging for acquisition, analysis, and visualization of the microscopy data.

Samples sectioned with a vibratome were dehydrated in a graded ethanol series (85, 96, and 100% – 1 h each). After dehydration, samples were stored in 100% ethanol at 4°C until further use. Longitudinal, 130 μm thick sections (depending on ovaries age) were made with a Leica series 1000S vibratome. Slices were collected in multiwell plates containing 70% ethanol, which avoided the curling of the slices. Afterward, slices were further dehydrated in a graded ethanol series (85, 96, and 100% – 1.5 h each). After dehydration, some samples were cleared in a graded series of absolute ethanol: methyl salicylate (1:1 and 1:3, v/v for 12 h), and then in 100% methyl salicylate for 24 h. Slices were analyzed using a Nikon Eclipse Ti S inverted microscope equipped with Nomarski’s differential interference contrast (DIC) optics, with appropriate filters for optimal viewing. Photographs were taken with a high-resolution Nikon DS-Fi1 5-megapixel cooled color digital microscope camera, coupled with a NIS-Element imaging for acquisition, analysis, and visualization of the microscopy data.

### Carpel/Ovule Culture and Isolation of Embryos

Rather than isolating embryos, we cultured individual ovary carpels, containing one ovule each. In this way, it was possible to culture immature zygotic embryos without physical injury and before they are fully formed, allowing their development up to cotyledonary stage inside the ovule. Previous studies in cassava ER were either conducted with directed crosses (hand-made pollinations) (Fregene et al., 1999; Akinbo et al., 2010) or with open pollinated populations (Biggs et al., 1986; Yan et al., 2014). In our work, the histological analysis for the determination of the embryo developmental stages through time was based on hand-pollinated cyathia. The number of pollinations required was limited and the procedure provided better-controlled conditions. However, the large number of ovules needed for the development of the ER protocol meant that controlled pollinations could not be made and thus, ER was based on open-pollinated inflorescences.

Bracts of the female cyathia usually open around noon or early afternoon (also referred herein as the date of anthesis or Day 0) and it is easy to predict those cyathia that will open within a day. Bracts usually close one DAA, and stigmas dehisce within 2–4 DAA. Cyathia were selected at anthesis, marked with plastic labels (identifying them and stating the date of anthesis) and left for natural open pollinations carried out by insects. Open-pollinated cyathia were harvested 7, 14, 21, 24, or 30 DAA. Collected cyathia were placed individually by treatment (days after anthesis) in zip-plug bags within a styrofoam cooler with refrigerant gel, and then transported to the laboratory within the next hour after collection for sample processing.

The cyathia were surface-sterilized with 70% ethanol for 1 min, followed by 1.5% sodium hypochlorite solution in water, with 3 drops of Tween 20 for 17 min, and rinsed four times with sterile distilled water. In the sterile environment, the cyathia were further dissected under a stereomicroscope (Nikon C-LEDS and cold-light Nikon NI-150). Pistils were cut off from cyathia just above the nectar glands. The stigmas were removed at the level of the style neck. Excised ovaries without nectar glands and without stigmas were cut longitudinally along the carpel walls in three sections containing one ovule each in each loculus. The carpels were cultured with the basal cut end on solid medium. Cultures were kept at 28–30°C in the dark. Once the ovules protruded through the carpel walls (usually about 3–4 weeks after the start of the culture), ovules were isolated and placed on fresh medium of the same composition with the adaxial side down on the medium. Ovules were subcultured every 4 weeks on fresh medium according to the medium sequence treatment as described below, until mature embryos (at advanced cotyledonary stage) naturally protruded through the ovule integuments or up to a total of 6 months. Mature embryos were subcultured to induce germination, and subsequently, to develop plants in a 12 h day/night photoperiod with 80–100 μmol.m^–2^.s^–1^ during the day.

### Culture Media for the Development of Mature Embryos

In a preliminary study, we evaluated the efficiency of the M6 medium used to rescue embryos at the earliest developmental stage ever reported for cassava (32 DAP, Yan et al., 2014). Culture of relatively young embryos requires proper osmotic adjustment of the culture medium (Haslam and Yeung, 2011). We compared the efficiency of the M6 medium, which consist of 1/2 MS (Murashige and Skoog, 1962) basal salts without vitamins, supplemented with GA3 1 mg/L and 2% sucrose, with the efficiency obtained using the same medium composition but with 3% sucrose. The M6 medium with 3% sucrose gave a better result (data not shown) for the rescue of mature embryos, and therefore, it was selected as control for our study. Medium salt concentration also has an effect on differentiation and maturation of cassava embryos developed *in vitro* (Groll et al., 2002). Hence, in this study the effect of half or full-strength of MS micro- and micronutrients supplemented with half or full-strength MS vitamins were also evaluated. Although ionic ammonium is a ready source of reduced nitrogen and is essential to embryo culture (Murray, 1988), at too high a concentration it can be toxic to embryo cultures (George and De Klerk, 2008). NLN contains a significantly lower level of nitrogen respect to MS. In the NLN medium, nitrogen is provided in the form of nitrate, and does not contain ammonium. NLN had also proven to induce embryogenesis in cassava (Perera et al., 2014a, b). Consequently, in addition to MS, the NLN micro- and micronutrients were also evaluated. In addition of the proper osmotic adjustment of the culture medium, culture of relatively young embryos requires as well as supplementation with vitamins, amino acids, and growth hormones. It is known that the optimal composition of the medium changes during embryo development (Haslam and Yeung, 2011). Therefore, for our experiments the stage of embryo development at the time of culture was taken into account.

Samples were divided in two groups. Group 1, consisted of cyathia collected at 7–14 DAA (containing embryos at the initial stage of development). Group 2, included cyathia collected at 21–30 DAA (containing embryos at globular stage and onward).

We applied various medium treatments taking into account the different nutritional requirements that the embryos may have according to their stage of development. Key parameters evaluated included: (a) basal medium micro- and macronutrients compositions and concentrations using as a base the MS (Murashige and Skoog, 1962) and NLN (Nitsch and Nitsch, 1969) media; (b) osmotic adjustment of the culture medium using different sucrose concentration (3, 8, and 13%); (c) supplementation of vitamins composition and concentrations using as base MS vitamins, (d) supplementation with amino acids, and growth regulators using as base previous reports on cassava embryo rescue (Yan et al., 2014) and optimal composition of medium for embryonic development of various species *in vitro* (Haslam and Yeung, 2011).

The detailed medium compositions tested are summarized in Tables 1, 2. These media were supplemented with 0.8 mg/L CuSO_4_ × 5H_2_O, which is a 32-fold increase in Cu with respect to the original MS medium. Increased amounts of Cu were reported to increase embryogenesis of cassava tissue culture *in vitro* (Schöpke et al., 1992; Danso and Ford-Lloyd, 2002). The M6 medium (Yan et al., 2014) with 3% sucrose was used as control.

**TABLE 1:** Initial medium compositions evaluated for rescuing cassava embryos.

**Components**	**Medium**					
	**M6^a^**	**1/2 MS^b^**	**1/2 NLN^c^**	**MSRE^b^**	**MSRE-V^b^**	**MS2^b^**
**Macro- and micro-elements (mg/L)**						
Ca(NO_3_)_2_ × 4H_2_O	–	–	360	–	–	–
CaCl_2_ × 2H_2_O	220	220	–	220	220	440
NH_4_ NO_3_	825	825	–	825	825	1,650
KNO_3_	950	950	62.5	950	950	1,900
MgSO_4_ × 7H_2_O	185	185	62.5	185	185	370
KH_2_PO4	85	85	62.5	85	85	170
H_3_BO_3_	3	3	5	3	3	6
MnSO_4_ × H_2_O	8	8	9.5	8	8	17
ZnSO_4_ × 7H_2_O	4.3	4.3	5	4.3	4.3	8.6
Na_2_MoO_4_ × 2H_2_O	0.13	0.13	0.125	0.13	0.13	0.25
CuSO_4_ × 5H_2_O	0.8	0.8	0.8	0.8	0.8	0.8
CoCl_2_ × 6H_2_O	0.025	0.025	0.0125	0.025	0.025	0.025
KI	0.42	0.42	–	0.42	0.42	0.83
Na_2_-EDTA	18.7	18.7	–	18.7	18.7	37.3
FeSO_4_ × 7H_2_O	13.9	13.9	–	13.9	13.9	27.8
**Vitamins, amino acids, other organics supplements (mg/L)**						
Nicotinic acid	–	0.25	2.5	0.25	0.5	0.5
Pyridoxine	–	0.25		0.25	0.5	0.5
Thiamine	–	0.05	0.25	0.05	0.1	0.1
Glycine	–	1	1	1	2	2
Myo-inositol	–	50	50	50	100	100
Biotin	–	–	0.025	–	–	–
L-glutamine	–	–	400	–	–	–
Folic acid	–	–	0.25	–	–	–
Glutathione	–	–	15	–	–	–
L-serine	–	–	50	–	–	–
2,4 D	–	–	–	–	–	2
NAA	–	–	–	0.01	0.01	–
Kinetin	–	–	–	–	–	0.5
GA3	1	–	–	1	1	1
Sucrose	30,000	1,30,000	1,30,000	30,000	30,000	80,000
Gellan Gum	3,000	3,000	3,000	3,000	3,000	3,000

*^*a*^Yan et al. (2014); ^*b*^Murashige and Skoog (1962); ^*c*^Nitsch and Nitsch (1969).*

**TABLE 2:** Medium composition for induction of mature embryos, germination, and development of viable plants from open-pollinated ovule cultures.

**Components**	**Medium**				
	**MS3**	**MS3-2**	**MS2 maturation**	**MS-BAP**	**4E**
**Macro- and micro-elements (mg/L) based on MS^a^**					
CuSO_4_ × 5H_2_O	0.8	0.8	0.8	0.8	0.8
** Vitamins, amino acids, other organics supplements (mg/L)**					
Nicotinic acid	2.5	2.5	0.5	0.5	–
Pyridoxine	1.2	1.2	0.5	0.5	–
Thiamine	10	10	0.1	0.1	0.5
Glycine	4	4	2	2	–
Myo-inositol	500	500	100	100	50
Biotin	0.2	0.2	–	–	–
Ca-pantotenate	0.2	0.2	–	–	–
Ascorbic acid	0.2	0.2	–	–	–
Riboflavin	0.4	0.4	–	–	–
L-proline	200	200	–	–	–
L-glutamine	400	400	–	–	–
Casein hydrolysate	150	150	–	–	–
2,4 D	2	0.5	–	–	–
NAA	–	–	1	–	0.02
Kinetin	–	–	–	–	–
BAP	2	–	–	0.45	0.04
GA3	1	1	1	–	0.05
Sucrose	80,000	80,000	20,000	20,000	20,000
Gellan gum	3,000	3,000			
Agar			5,000	4,500	4,000

*^*a*^MS medium (Murashige and Skoog, 1962).*

For the first set of experiments, the medium compositions evaluated according to the two groups of embryo stage of development at the moment of *in vitro* culture were:

**Group 1** (7–14 DAA): M6, MSRE, MSRE-V, and MS2 media (Table 1).
**Group 2** (21–30 DAA): M6, MSRE-V, 1/2 MS, and 1/2 NLN media (Table 1).

**FIGURE 1:**
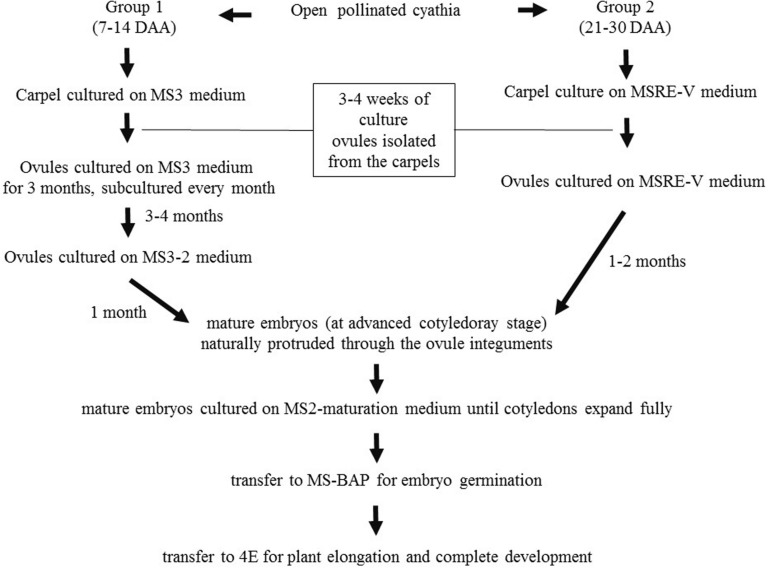
Flowchart of medium sequence for induction of mature embryos (cotyledonary stage of development), germination, and development of viable plants from open-pollinated ovules cultures. Group 1, consisted of cyathia collected 7–14 DAA, which contained embryos at the initial stage of development. Group 2, included cyathia collected 21–30 DAA, which contained embryos at globular stage or onward. The carpels were cultured with the basal cut end on solid medium. Cultures were kept at 28–30°C in the dark. Once the ovules protruded through the carpel walls (usually about 3–4 weeks after culture initiation), ovules were isolated and placed on fresh medium of the same composition with the adaxial side down on the medium. Ovules were subcultured every 4 weeks on fresh medium according to the medium sequence treatment, until mature embryos (at advanced cotyledonary stage) naturally protruded through the ovule integuments or up to a total of 6 months. Mature embryos were subcultured to induce germination, and subsequently, to develop plants in a 12 h day/night photoperiod with 80–100 μmol.m^– 2^.s^– 1^ during the day.

Based on the results obtained during the first set of experiments, later experiments were conducted to evaluate other medium compositions (Table 2), aiming at increasing the induction of cotyledonary embryo formation from Group 1. Carpels from cyathia collected at 7–14 DAA (Group 1), were first cultured on MS3 medium (Table 2 and Figure 1). After 4 weeks of culture, the ovules were isolated from the carpels, and transferred onto fresh MS3 medium, and grown under the same environmental conditions. The ovules were subcultured on the same medium for 3 months. After this process, ovules were transferred to MS3-2 medium (Table 2 and Figure 1) until mature embryos were developed. In contrast to Group 1, carpels from cyathia collected at ≥21 DAA (Group 2), were cultured on MSRE-V medium (Table 1) instead of on MS3 medium (Figure 1). After 4 weeks of culture, the ovules were isolated from the carpels, and transferred onto fresh MSRE-V medium, and grown under the same environmental conditions until mature embryos were developed. For both groups, once mature embryos (at advanced cotyledonary stage) naturally protruded through the ovule integuments, they were transferred to the medium sequence for germination and, subsequently, for conversion into plants as described in the section below.

### Embryo Germination and Conversion Into Plants

Cotyledonary embryos formed on MS3-2 medium from Group 1, or on MSRE-V from Group 2, were sub-cultured on MS2-maturation medium (Table 2 and Figure 1). Cultures were kept at 28–30°C in the dark for 2 weeks or until the cotyledons expanded fully. Embryos with expanded cotyledons, were transferred on MS-BAP medium (Table 2 and Figure 1) for germination and incubated at 28–30°C in a 12 h day/night photoperiod with 80–100 μmol.m^–2^.s^–1^, for further development. Once the first true leaves expanded and roots were formed, plants were transferred on 4E medium (Roca, 1984) (Table 2 and Figure 1) for plant elongation and full development, and incubated at 28–30°C in a 12 h day/night photoperiod with 80–100 μmol.m^–2^.s^–1^.

### Adaptation of Developed Plants From *in vitro* to Greenhouse Conditions

Fully developed plants *in vitro*, were acclimatized inside the culture room chamber for 2 weeks by eliminating the food wrap closure, and then loosening the *in vitro* culture jar cap. Plants were removed from the culture vessel, and the remaining medium was washed out from the roots with running water. Plants were transferred in the same vessel with liquid 4E medium without sucrose for 8 days. Plants were then transferred into trays adapted for hydroponic culture system (Castañeda-Méndez et al., 2017). After profuse root development, plants were transferred to sterile soil for further development in the screen house.

### Experimental Design and Statistical Analysis

The experimental design for the evaluation of induction of mature embryo formation was set up with nine replicates, including five embryo developmental stages (7, 14, 21, 24, and 30 DAA), and various *in vitro* culture media according to the Group. For Group 1 (7–14 DAA), a total of six media were evaluated for the induction of mature embryo formation. These media were M6, MSRE, MSRE-V, MS2, MS3, and MS3-2 media. For Group 2 (21–30 DAA), a total of four media were evaluated for the formation of mature embryo: M6, MSRE-V, 1/2 MS, and 1/2 NLN media. Each replicate consisted of one petri dish with fifteen carpels (with one ovule each). Therefore, 135 ovules were cultured for each stage per media combination, requiring the dissection of more than 3,000 ovules.

The cultures were evaluated by assessing changes in the ovule size and color (including degeneration of integuments and other tissues), occurrence of mature embryo formation, embryo germination and plant development. Photographs of cultures were taken with a high-resolution Nikon DS-Fi1 5-megapixel cooled color digital microscope camera, coupled with a NIS-Elements imaging for acquisition, analysis, and visualization of the microscopy data. The cultures were evaluated by the frequency of mature embryo formation (number of ovules with mature embryos/100 ovules), and the frequency of plant formation (number of plants developed from mature embryos rescued and cultured/100 embryos).

Marascuilo’s test for testing equality of several proportions was performed for mature embryo formation and plant formation. In all cases, the analyses were performed using the SAS statistical program, software version 9.4 (SAS, 2020). To maximize the power of the analysis, all treatments were compared with each other at a confidence level of 0.05. In the figure and table reporting the results of these analyses, different letters were assigned to the treatments showing a significant difference at a *P*-value = 0.05 according to the contrast output.

## Results

### Stage of Embryo Development in Hand-Pollinated Ovules Collected at 1–30 DAA

**FIGURE 2:**
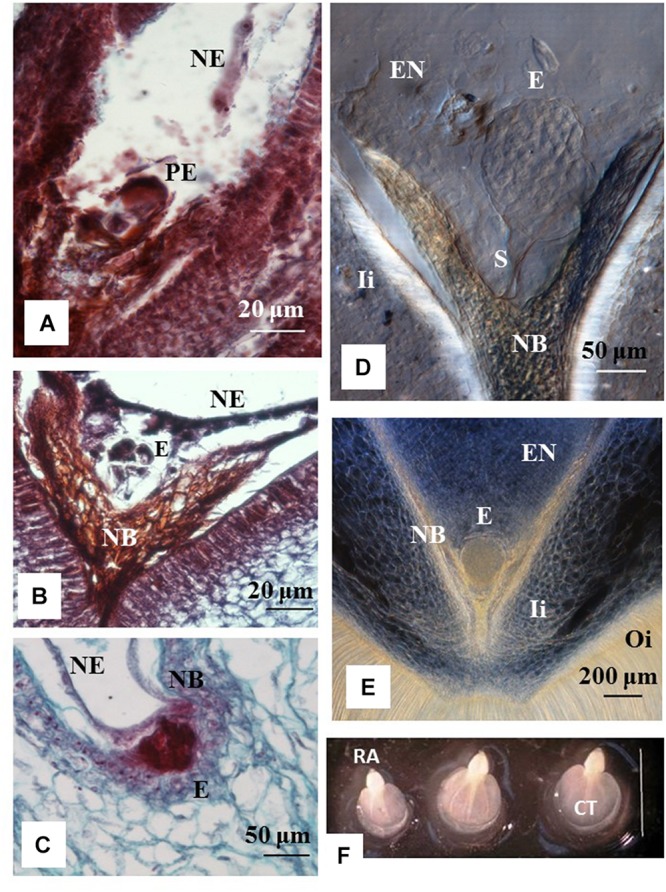
Stage of development of cassava zygotic embryos from hand pollinated ovules. Histology sections of ovules from cyathia **(A)** collected 7 days after anthesis (DAA); **(B)** 14 DAA; **(C)** 21 DAA; **(D,E)** 24 DAA; and **(F)** 30 DAA. **(A)** Shows a pro-embryo stage of development. The first signs of nucleated endosperm are also noted at this stage. **(B)** Show an embryo at pre-globular stage, nucellar tissue degradation and clear signs of endosperm formation. **(C)** Shows an embryo at early globular stage. **(D,E)** Correspond to embryos at proper globular stage. At this stage of development, the endosperm is apparent. **(D)** The embryo suspensor is visible between the embryo and the nucellar beak. **(E)** The nucellar beak shows significant degradation compared to earlier stages, the outer and inner integuments are thickening, and the endosperm is fully formed. **(F)** Correspond to dissected fully formed cotyledonary embryos. The cotyledons and the root apex are visible in each embryo. Bar = 3 mm. **(A,B)** Correspond to longitudinal sections of 10 μm thick and **(C)** to longitudinal sections of 4 μm thick. **(A–C)** The sections were processed with microtome, and stained with Safranin-O and Fast Green. Photos were taken at 40× using a light microscope. **(D,E)** Correspond to longitudinal sections of 130 μm thick, which were processed with vibratome. **(D)** The section was cleared with methyl salicylate and observed with Nomarski’s differential interference contrast (DIC) optics. Photos were taken at 20× in **(D)** and at 10× in **(E)**. PE, pro-embryo; E, embryo; NE, nucleated endosperm; Ii, inner integument; Oi, outer integument; EN, endosperm; S, suspensor; RA, root apex; CT, cotyledon.

**FIGURE 3:**
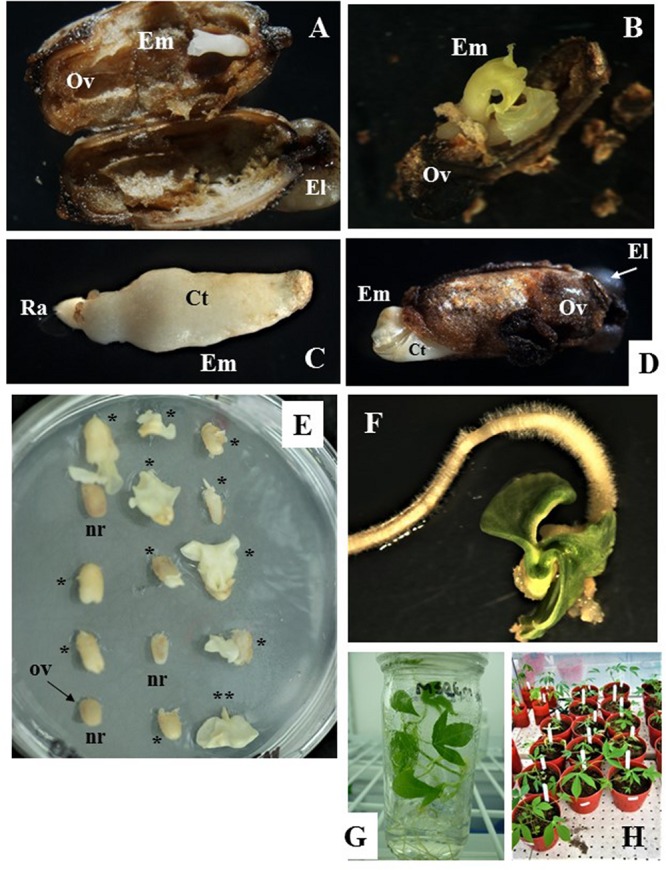
Zygotic embryo development and rescue, and recovery of full grown plants from open pollinated ovule cultures. **(A,B)** Show cotyledonary embryo formed in ovules from cyathia collected 7 days after anthesis (DAA) and cultured on MS3 medium for 3 months and then sub-cultured on MS3-2 medium for 1 month. In **(A)** embryo shows fused cotyledons, whereas in **(B)** cotyledons are fully formed and are expanding. **(C,D)** Correspond to cotyledonary embryos formed in ovules from cyathia collected 14 DAA. In **(C)**, the embryo shows fused cotyledons covered by nucellus tissue and the root apex is clearly identified. Embryo was formed on MS2 medium. In **(D)**, the embryo is fully formed and it is protruding through the ovule wall. In this case, the embryo was formed on MS3 medium for 2 months, and then sub-cultured on MS3-2 medium for 1 month. **(E)** Show ovules from cyathia collected 30 or 21–24 DAA, and cultured on MSRE-V for 1 or 2 months, respectively. Different responses are noted between ovules collected at the same DAA and cultured in the same replicate (petri dish). Few of the ovules show no signs of response (nr), whereas most of the ovules show cotyledonary embryos protruding through the ovule integuments, and with different level of development (*). (**) Corresponds to a mature embryo (at advanced cotyledonary stage) showing fully expanded cotyledons and the root apex. **(F)** Shows a mature embryo germinating on MS-BAP medium. **(G)** Shows a fully developed plant, growing on 4E medium in the culture room at 28–30°C in a 12 h day/night photoperiod with 80–100 μmol.m^–2^.s^–1^. **(H)** Corresponds to plants from embryo rescue growing in the greenhouse. Em, cotyledonary embryo; Ov, ovule integuments; El, elaiosome; Ct, cotyledons; Ra, root apex.

Histology analyses indicated that ovules from 1 to 3 DAA showed increased accumulation of starch granules at the embryo sac (data not shown). The first cell divisions were noted in ovules at 7 DAA, which contained pro-embryo stage of development (Figure 2A). The first signs of nucleated endosperm were also noted at this stage (Figure 2A). At 14 DAA embryos were still at the initial stage of development and few divisions of the egg cell were clearly distinguished. Embryos were at the pre-globular stage, showed nucellar tissue degradation and early signs of endosperm formation (Figure 2B). The first embryos at the early globular stage were observed at 21 DAA (Figure 2C). Proper globular stage was clearly found at 24 DAA (Figures 2D,E). At this stage of development, the endosperm was apparent and the embryo suspensor was clearly identified (Figure 2D). The nucellar beak showed significant degradation compared to earlier stages, the outer and inner integuments were thickening, and endosperm was formed (Figure 2E). Beyond 24 DAA, embryos continued to mature, reaching the cotyledonary stage (Figure 2F). These embryos were ready for germination once they were rescued and cultured *in vitro* (Figure 3F).

### Morphological Changes Associated With the Formation and Development of the Zygotic Embryo Inside the Ovules in Culture

After isolation from the carpels, ovules cultured *in vitro* often increased in size and showed external morphological changes in color, which were associated with the formation and development of the zygotic embryo inside the ovule. The external ovule integuments turned brown (Figures 3A,B,D) and, in some cases, dehisced as the mature embryo developed and increased in size (Figure 3D). Ovules that did not respond to the *in vitro* culture, did not develop beyond the status at the moment of excision from the carpel (note: ovules are labeled as “*nr*” in Figure 3E). The ovules eventually shrank and were empty inside.

### Effect of Medium Composition on the Formation of Mature Embryos and the Conversion of Mature Embryos Into Plants

Taking into account the different nutritional requirements of the embryos according to their stage of development, our experiments were conducted to determine the best medium composition, allowing zygotic embryo formation and its maturation *in vitro* from open-pollinated ovule cultures. Experiments were also designed to define the best conditions to further sustain embryo development up to germination and recovery of fully developed plants. Various medium compositions were tested. The M6 medium used for embryo rescue of cassava 32–36 DAA as reported by Yan et al. (2014) was used as control. Several medium composition modifications were introduced, based on what it is known about the requirements for younger and older embryos, as well as on the information obtained during the course of this work, regarding the stage of development of the embryos at the time of ovule collection and the ovule culture responses. The effectiveness of the media evaluated was analyzed based on the percentages of embryos reaching the advanced cotyledonary stage (mature stage) after ovule culture; and the frequency of rescued embryos that germinated and converted into fully developed plants. In line with other reports, our results also suggest that optimal medium composition for the complete development of embryos in ovule cultures, differed depending on the stage of embryo development at the moment of excision from the ovary.

In order to understand the different medium requirements according to the stage of embryo development at the initiation of the culture, experiments were separated in two groups. Group 1 (7 and 14 DAA), included embryos at the initial stage of development prior to the globular stage, whereas Group 2 (21, 24, and 30 DAA), consisted of embryos that were at least at the globular stage or onward at the time of the culture.

For the first set of experiments, the response of Group 1 was evaluated on M6, MSRE, MSRE-V, and MS2 media (Table 1). Ovules of 7 DAA, which contained pro-embryos at the time of culture, did not respond at all on any of these media. In contrast, when 7-DAA ovules were cultured on richer and complex media such as MS3 medium and then sub-cultured on MS3-2 medium (Table 2), embryos developed through the cotyledonary stage after a total of 4 months of culture (Figure 3A,B). These mature embryos with fully expanded cotyledons, germinated after rescued, and converted into viable plants (Figure 3G).

Ovules collected 14 DAA, contained embryos at pre-globular stage at the time of culture, also responded differently to medium composition. The higher the content of macro- and microelements, vitamins and other organics supplements, the better the response. MS2 medium (Table 1) significantly induced (*P* = 0.05) the highest formation of mature embryos (average 16.3%) followed by the MSRE-V medium (10.8%) after 2 months of culture. The lowest response was noted on MSRE medium (3.9% formation of mature embryos) and M6 medium (3.0% formation of mature embryos). However, none of these media induced the conversion of embryos into plants. The mature embryos had fused cotyledons that did not expand (Figure 3C), and embryos were unable to germinate. As in the case of 7-DAA ovules, 14-DAA ovules also showed an improved response when cultured on MS3 medium and then sub-cultured on MS3-2 medium (Table 2). After a total of 3 months of culture using this media sequence, the mature cotyledonary embryos protruded through the chalazal end-side of the ovule, showing fully formed cotyledons (Figure 3D), and were ready for germination (Figure 3F).

Histological analysis of Group 2 indicated that embryos were at pre-globular stage at 21 DAA, proper globular stage at 24 DAA, and early cotyledonary stage at 30 DAA. Taking into account that the embryo development is not always synchronous between loculi of the same cyathia, the data from 21 and 24 DAA were combined to maximize the power of the analysis. For the first set of experiments, the response of Group 2 was evaluated on M6, MSRE-V, 1/2 MS, and 1/2 NLN media (Table 1).

**FIGURE 4:**
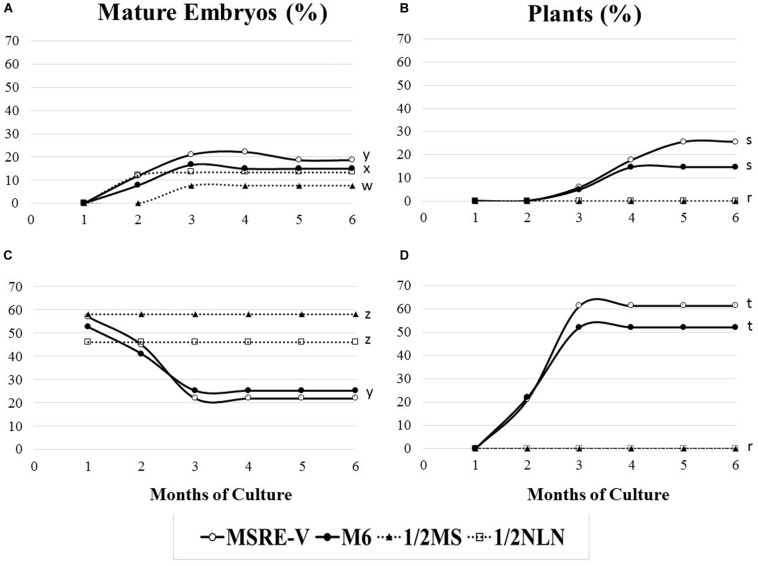
Response to mature-embryo formation in ovule cultures and embryo conversion into fully developed plants from open pollinated cassava cyathia. The response was evaluated by the frequency of mature embryo formation (number of ovules with mature embryos/100 ovules), and the frequency of plant formation (number of plants developed from mature embryos rescued and cultured/100 embryos). **(A,B)** Correspond to ovules from cyathia collected 21–24 days after anthesis (DAA). Data from 21 and 24 DAA were combined, since the embryo formation might not be synchronous between loculi of the same cyathia. **(C,D)** Show response of ovules from cyathia collected 30 DAA. For all the treatments, ovules were isolated and cultured after 4 weeks of culture in the carpels and kept at 28–30°C in the dark. Ovules were subcultured, according to the medium sequence treatment, until mature embryos (at advanced cotyledonary stage) naturally protruded through the ovule integuments or up to a total of 6 months. Mature embryos were subcultured to induce germination and subsequently, to develop plants in a 12 h day/night photoperiod with 80–100 μmol.m^–2^ s^–1^ during the day. Marascuilo’s test for equality of several proportions was performed for mature embryo formation and plant formation. To maximize the power of the analysis, all treatments were compared with each other at a confidence *P*-value = 0.05. Values with identical letters indicate no significant difference. Only statistical values at 6 months of culture are shown in order to facilitate legibility of results.

The composition of the medium and the stage of development at the time of culture (DAA) were key factors for both the efficiency of formation of mature embryos (advanced cotyledonary stage) and their conversion into plants (Figure 4 and Table 3). Ovules cultured at 30 DAA showed the highest formation of mature embryos (between 45 and 57%, Table 3) 1 month after culture (Figure 4C). In contrast, the formation of mature embryos in ovules cultured at 21–24 DAA, was delayed for up to 2 months in comparison with 30 DAA ovules (Figures 4A,C). Between 13 and 22% of ovules cultured at 21–24 DAA significantly produced fewer mature embryos (about 30% less) respect to 30 DAA ovules (Table 3). As the time of culture progressed, there was a significant and clear reduction in the number of mature embryos due the conversion of the fully formed embryos into plants (Figures 4B,D). This trend was more clearly seen with 30 DAA cultures on MSRE-V and M6 media (Figures 4C,D). The highest percentage of plants (52–61%) were noted for 30 DAA after 3 months of culture (Figure 4D and Table 3). In the case of 21–24 DAA cultures, the highest conversion into plants (15–25%, Table 3) was obtained at 5 months after culture (Figure 4B). No plants were obtained on 1/2 MS nor on 1/2 NLN media, independently of the stage of development (DAA) at the time of culture initiation (Figures 4B,D and Table 3). The highest conversion of embryos into plants was obtained on MSRE-V medium for both, 21–24 and 30 DAA cultures (Figures 4B,D and Table 3). Cultures at 30 DAA showed a 61% efficiency in plant conversion on MSRE-V medium and 52% on M6 medium. This is about threefold higher than the response obtained on the same media with cultures at 21–24 DAA (Figures 4B,D and Table 3). No changes in the pattern of response was noted after 5–6 months of culture (Figures 4A–D), reason why the experiments were terminated at this time.

**TABLE 3:** Induction of mature embryos (cotyledonary stage of development) from open-pollinated ovule cultures and conversion of mature embryos into viable plants.

**DAA^z^**	**Culture medium^y^**	**Ovules^x^**	**Mature Embryos^w^**	**% Mature Embryos^v^**	**Plants^u^**	**% Plants^t^**
21–24	MSRE-V	270	61	22.6 b	15	24.6 b
21–24	M6	270	47	17.4 c	7	14.9 b
21–24	1/2MS	270	20	7.4 d	0	0.0 d
21–24	1/2NLN	270	36	13.3 c	0	0.0 d
30	MSRE-V	135	77	57.0 a	47	61.0 a
30	M6	135	71	52.6 a	37	52.1 a
30	1/2MS	135	79	58.5 a	0	0.0 d
30	1/2NLN	135	62	45.9 a	0	0.0 d

*Marascuilo’s test for essaying equality of several proportions was performed for mature embryo and plant formations. To maximize the power of the analysis, all treatments were compared with each other at a confidence level of 0.05. Values with identical letters indicate no significant difference according to the contrasts output. ^*z*^Days after pollination. To maximize the power of the analysis, data from 21 and 24 DAA were combined, since the embryo formation might not be synchronous between loculi of the same cyathia. ^*y*^Medium composition according to Table 1. ^*x*^Ovules cultured per DAA per medium. ^*w*^Total number of cotyledonary embryos formed and rescued from the ovule cultures. ^*v*^Frequency of mature embryo formation (number of ovules with mature embryos/100 ovules). ^*u*^Total number of plants developed from cotyledonary embryos rescued and cultured. ^*t*^Frequency of plant formation (number of plants developed from the mature embryos rescued and cultured/100 embryos).*

### Optimizing Embryo Rescue and Conversion Into Plants According to the DAA for Ovule Culture

The results presented above suggest that the optimal sequence of media compositions to rescue cassava embryos varies and depends highly on their stage of development at the time of culture. In short, the earlier the stage of the embryo development at the time of the carpel culture, the richer and more complex the medium composition required for the initial phases of the embryo rescue. Once the mature embryo is fully formed (advanced cotyledonary stage), the protocol to recover fully develop plants is similar for all the cultures, independently of the DAA as summarized in Figure 1 and described in detail below.

Carpels from 7–14 DAA should be cultured initially on MS3 medium (Table 2 and Figure 1). Once the ovules protruded through the carpel walls, after 3–4 weeks of culture, they were isolated and placed on MS3 fresh medium and grown under the same environmental conditions. Ovules should be sub-cultured on the MS3 medium every 4 weeks for about 3 months. After this time, ovules should be transferred to MS3-2 medium (Table 2) until the formation of mature embryos at advanced cotyledonary stage. Embryos naturally protrude though the ovule integuments when they are fully formed (Figures 1, 3D,E). In contrast, carpels from 21 to 30 DAA should be cultured on MSREM-V medium (Table 1), and once the ovules protrude through the carpel integuments, the ovules should be sub-cultured on fresh MSREM-V medium for another month until mature embryos develop (Figure 1).

Upon reaching the cotyledonary stage, the embryos are isolated from the remaining ovule tissue to allow further development on the same medium (MS3-2 or MSREM-V according to the DAA). Once the mature cotyledons are fully expanded (note: embryo tagged as (**) in Figure 3E), embryos are transferred directly on MS maturation medium, then on MS-BAP medium for germination (Table 2 and Figure 1). Embryos fully formed at advanced cotyledonary stage, germinate with profuse root growth and expand the first true leaves on MS-BAP medium (Figure 3F). Subsequently, plant elongates, develops fully and grows into healthy green plants (Figure 3G) on 4E medium (Table 2 and Figure 1). Afterward, plants are acclimated in the culture room, and then transplanted into soil, and grown in the screen house (Figure 3H).

With this protocol (Figure 1), the rescue of mature embryos might take about 1, 2, 3, or 4–6 months after the initial date of culture of isolated ovules collected at 30, 24–21, 14, or 7 DAA, respectively. Likewise, following this protocol (Figure 1), it might be possible to obtain plants with an efficiency ≥80% from cultures at 30 DAA; 30–50% at 21–24 DAA; 10–20% at 14 DAA; and 0–5% at 7 DAA, respectively.

## Discussion

This study documents the cassava embryo development during the first 4 weeks after anthesis. Information that had not been taken into account for the development of ER protocols for cassava so far. Prior to culturing, it is important to know the stage of development of the embryos in order to tailor the medium composition to be used. The physical and chemical environments surrounding the zygotic embryo *in ovulo* are very complex (Steeves and Sussex, 1989; Yeung et al., 2001). For successful *in vitro* culture of small proembryos, the best approach is to simulate an environment as close to *in ovulo* conditions as possible (Haslam and Yeung, 2011). Our results suggest that cassava embryos and the endosperm develop slowly during the first 3 weeks after anthesis. Moreover, our results indicate that in cassava, proembryos are still in development during the first 2 weeks after anthesis. The conditions needed to support the proembryos development up to maturity, as expected, were different from those used to rescue embryos at 32 DAA or beyond.

Here we present a different approach for embryo rescue in cassava compared with previous works. Rather than isolating embryos, individual ovary carpels (containing one ovule each), were cultured. This approach made possible to successfully culture immature zygotic embryos before they were fully formed. This type of culture would support the development of the proembryos inside the ovules, avoiding their physical damage due to handling, preserving the chemical conditions surrounding the proembryos, and facilitating their nurture via exogenous supply from the culture medium. This method of culture, in combination with an optimized rich culture medium composition sequence, allowed rescuing embryos at very early stages of development (proembryo or pre-globular) collected 7 or 14 DAA. More importantly, the protocol also sustained embryo maturation *in vitro* through the cotyledonary stage and, subsequently, their germination and recovery of fully developed plants. The cotyledonary embryos, when fully formed, either emerged spontaneously or had to be excised from the ovule tissue to allow their germination. Embryos that had fully expanded cotyledons were mature enough for root formation (germination), the expansion of the first true leaves, and subsequently, for recovering fully grown plants. These results are in compliance with those obtained by Monnier (1984). The globular embryos of *Capsella bursa-pastoris* of less than 50 μm in size that grow in ovules cultured *in vitro* show much better survival than when they are inoculated singly onto the medium.

The culture medium has a key effect on the response of rescued embryos. In spite of the fact that there are various media commonly use, the majority of them had not been tested rigorously for most species (Haslam and Yeung, 2011). However, there are some general requirements that can be used as guidance when optimizing medium compositions according to the developmental stage of interest. Immature zygotic embryos are heterotrophic and have more complex nutritional requirements, while more mature embryos are autotrophic, therefore, can be grown in a simpler inorganic macro and micro-elements compositions. The culture of immature zygotic embryos requires proper osmotic adjustment of the culture medium, as well as supplementation with vitamins, amino acids, and growth hormones (Yeung et al., 2001).

Several articles provide comprehensive information on embryo nutrition and discuss the construction of media for embryo growth *in vitro* (Murray, 1988; George et al., 2008). It has been concluded that reduced nitrogen strongly influences embryo growth in culture. Ionic ammonium is a ready source of reduced nitrogen and is essential to embryo culture (Murray, 1988), however, at too high a concentration it can be toxic to cell and embryo cultures (George et al., 2008). Amino acids are readily absorbed and can be used directly as a source of nitrogen. The addition of amino acid mixtures, such as casein hydrolysate, or specific amino acids, such as glutamine, and other amino acids have a positive influence on embryo culture. Likewise, in our work, the supplementation of the MS3 and MS3-2 media with casein hydrolysate, glutamine and proline allowed the rescue of proembryo or pre-globular embryos.

The evaluation of media was based on the results from our study on embryo development during the first 4 weeks after anthesis, and took into consideration that the largest embryo abortion in cassava occurs during the first 2 weeks after anthesis (Yan et al., 2014). Different sequences of various medium composition were tested. The aim was to induce a complete development of zygotic embryos up to the advanced cotyledonary stage (mature embryos), that are ready for germination and for developing plants. Our results clearly indicated that the response is highly dependent not only on the stage of development of the embryos at the time of rescue, but also on the composition of the culture medium. As expected, the more advanced the stage of development, the simpler the medium composition needed and the easier the recovery of fully developed plants. The earlier stages of embryo development required richer medium composition. The M6 medium used by Yan et al. (2014) for embryo rescue of cassava 32–36 DAP, failed to induce embryo maturation, nor the formation of plantlets and plants from ovules collected at 7 and 14 DAA, which contained pro-embryos and pre-globular stage of embryo developments. The highest response from stages younger than the globular stage was induced, when medium contained complete MS macro and micro salts. It was supplemented with stronger and higher concentration of auxin (2 mg/L 2,4-D), a cytokinin (0.5 mg/L BAP) and gibberellin (1 mg/L GA3), intermediate level of sucrose (8%), higher content of organic acids, and source of amino acids, as in MS3 medium. Responses decreased when sucrose was reduced to 3% and 2,4-D was replaced by NAA as in MSRE-V medium. Of the media evaluated, MS3 medium used as the initial medium in combination with the MS3-2 medium, induced fully embryo formation from younger stages of zygotic embryos prior to the globular stage (ovules at 7 DAA and DAA), that converted into fully developed plants.

In contrast to the earlier stages of development, ovules ≥ 21 DAA, (embryos that are at least at globular stage), have higher response on medium with half strength of MS salts but complete MS vitamins (MSREM-V). Once embryos were fully developed (cotyledonary stage), it was possible to easily obtain plants. Results also suggest that high levels of sucrose (as high as 13% as in 1/2NLN and 1/2 MS media), although sustaining embryo development from ovules collected at ≥ 21 DAA, did not induce embryo maturation for further plant recovery. Embryos at globular (21–24 DAA) stage of development, showed higher frequency of embryo maturation and plant formation when cultured on media containing growth regulators. The addition of NAA and GA3 increased full development of plants. In contrast, ovules containing embryos at early cotyledonary stage at the moment of culture (30 DAA) were able to continue growth and development (advanced cotyledonary stage) independently of growth regulators. However, once the cotyledonary embryos are formed from 30 DAA cultures, the addition of growth regulators significantly promoted a faster maturation, germination and conversion into plants of the embryos. A similar trend was also noted with ovules cultured at earlier DAA age.

Our results are in accordance with studies in other species. It has been demonstrated that auxin has a key regulatory function and is essential to axis establishment at the proembryo stage (Hamann, 2001). Additionally, culture of relatively young embryos requires proper osmotic adjustment of the culture medium, as well as supplementation with vitamins, amino acids, and other growth regulators. Steeves and Sussex (1989) demonstrated that zygotic embryos develop in an environment with highly negative water potential. Higher concentration of sucrose improved growth of the zygotic embryo *in vitro*. In addition to sucrose, the majority of media components also contribute to the total water potential of the medium. The concentration of sucrose used must be tested and optimized. The negative osmotic environment may also have a morphogenetic role and appears to regulate precocious germination of maturing embryos (Yeung et al., 2001; Thorpe et al., 2008). Young embryos require a high concentration of sucrose as an osmoticum to prevent precocious germination, as well as higher calcium concentration, which has been observed to have a role in protecting embryos during development. Young embryos also require low concentrations of selected minerals that can be toxic at higher concentrations, as they can be especially sensitive to their negative effects. They also benefit from a high concentration of amino acids, as they lack enzymes necessary for nitrate catabolism. As proembryos develop *in vitro*, their osmotic and nutritional requirements change. In general, a more positive water potential is favored as the embryos mature, and their nutritional requirements become less stringent relative to those of the proembryo (Yeung et al., 2001). Conversely, media designed for older embryos are generally characterized by lower concentrations of sucrose and amino acids. Older embryos grown on medium containing 13% sucrose as an osmoticum tended to grow larger and had a lower percentage of conversion into plants relative to their younger counterparts (Ilic-Grubor et al., 1998; Yeung et al., 2001).

In the present study, we proposed the use of ovule culture as a method of choice for cassava ER rescue. This methodology allowed the culture and rescue of embryos at the first stages of development prior to the globular stage. As expected, the earlier the ovules were isolated (DAA) and cultured, the longer it took to recover fully mature embryos. It took about 1 month of *in vitro* culture to recover mature embryos from ovules collected at 30 DAA, whereas it took 2, 3 or 4–6 months to recover mature embryos *in vitro* from 24, 21, and 7–14 DAA ovule cultures, respectively. Following this protocol, it might be possible to obtain plants with an efficiency of about 80% from cultures at 30 DAA; 30–50% at 21–24 DAA; 10–20% at 14 DAA; and 0–5% at 7 DAA, respectively. Although a significant progress was made, still there is work to be done to increase further the efficiency of response of ER during the first 2 weeks after pollination for a standard protocol of practical use in cassava breeding, which usually requires large numbers of plants. However, the information generated may prove useful to design new studies for a better understanding of the requirements of young cassava embryos and develop more efficient protocols.

In addition of using carpel/ovule culture and rich medium composition for culturing these young embryos as described herein, other approaches might be useful to explore. In order to accommodate the nutritional requirements of younger embryos, other culture systems could be evaluated. Monnier (1995) proposed a system for culturing *Capsella bursa-pastoris* proembryos consisting of two concentric rings of solid media. Medium suitable for more mature embryos surrounds a central section of young embryo medium in the plate where young embryos are cultured. Another approach is the use of a double-layered medium for the culture of zygote and proembryos, like the one being use for *Zea mays* study (Mol et al., 1995). However, these requirements are also highly dependent on the genotype and the growing conditions of the donor plants, which affect the vigor and the physiology of the tissues. Cassava is well known for its recalcitrance and high genotype-dependent response to various *in vitro* culture techniques (Chavarriaga-Aguirre et al., 2016). So far, the potential of using biotechnology to improve cassava has been significantly dependent on the success in optimizing and adjusting the various technologies specifically to this crop. Progress and lessons learned from other crops have so far been of limited use. Therefore, it is necessary to study further the culture conditions to optimize them according to nutritional requirements and growth regulator levels suitable for cassava.

Several studies for various species have shown the similarity of somatic embryoids and zygotic embryos in terms of morphological, histological, biochemical, and physiological aspects (Winkelmann, 2016). Hence, the vast knowledge accumulated to improve somatic embryogenesis in cassava may be worthwhile testing for cassava ER. Our results indicate that the highest response from stages younger than the globular stage was induced, when medium was supplemented with stronger and higher concentration of auxin and cytokinin. Different concentrations and stronger growth regulators could be evaluated. Recently, it has been demonstrated that meta-topolin stimulates *de novo* shoot organogenesis and plant regeneration from somatic embryogenesis in cassava (Chauhan and Taylor, 2018). The combination of meta-topolin with 2,4-D in a first culture medium, followed by culture on elevated concentrations of meta-topolin alone, significantly increased shoot regeneration. Another work toward the optimization of somatic embryogenesis in cassava suggest the need to evaluate the use of other synthetic auxins different from 2,4-D, such as picloram or dicamba (Danso and Elgba, 2017). The use of 2,4-D for embryo initiation often results in low frequency of primary embryo production as well as poor conversion into plants due to lack of root primordium (Danso and Elgba, 2017). The application of new generation of growth regulators such as oligosaccharides, jasmonate, polyamines, brassinosteroids, and phloroglucinol, had also proved to be useful for primary somatic embryos induction in many plant species (Danso and Elgba, 2017). The use of temporary immersion systems had demonstrated to increase somatic embryogenesis in cassava (Chavarriaga-Aguirre et al., 2016). Liquid medium had been used to increase the embryo rescue efficiency for culture of immature zygotic embryos in recalcitrant species such as coconut (Muhammed et al., 2013) and mango (Pérez-Hernández and Grajal-Martí, 2011). Abscisic acid (ABA) pretreatment in embryo maturation medium had proved to increase the conversion of cassava somatic embryos into plants (Danso and Elgba, 2017). Likewise, ABA has a similar effect on the maturation of immature zygotic embryos of cacao increasing their conversion into plants (Creaser-Pence, 1992). According to Chavarriaga-Aguirre et al. (2016), although important improvement has been obtained, the germination of cassava somatic embryoids ranges from 40 to 80%, indicating the need for further improvement. Therefore, conversely, the knowledge generated on culture of zygotic embryos and conversion into plants may be used as reference as well, to improve further somatic embryogenesis in cassava.

ER may play an important role in cassava modern plant breeding, allowing the development of hybrids from broad crosses and to facilitate the introgression of traits from inter-specific hybridizations. In addition, besides of its practical applications, zygotic embryo culture can be an excellent experimental system for pure scientific research. Understanding embryo growth provides a better theoretical understanding of plant growth and development in general, especially during the unique period when tissues, organs, and apical meristems are being established. A surge of information published in recent years concerning zygotic embryo development can attest to growing interest in the field, and in experimental studies on the structural functions, hormonal roles, and the molecular biology of embryo development, embryo culture complements *in ovulo* studies of zygotic embryogenesis. This work is a contribution to advance our understanding of embryo development in cassava. The knowledge gained may have direct practical implications to cassava breeding and the use of biotechnology to improving cassava.

## Data Availability Statement

All datasets generated for this study are included in the article.

## Author Contributions

ZL designed the research. GR, MB and ET researched under the supervision of ZL. All authors analyzed the data. ZL wrote the manuscript. All authors read and approved the final manuscript.

## Conflict of Interest

The authors declare that the research was conducted in the absence of any commercial or financial relationships that could be construed as a potential conflict of interest.
